# A novel risk-based decision-making paradigm

**DOI:** 10.3389/fnbeh.2014.00045

**Published:** 2014-02-17

**Authors:** Pedro Morgado, Fernanda Marques, Miguel B. Silva, Nuno Sousa, João J. Cerqueira

**Affiliations:** ^1^School of Health Sciences, Life and Health Sciences Research Institute (ICVS), University of MinhoBraga, Portugal; ^2^ICVS-3Bs PT Government Associate LaboratoryBraga/Guimarães, Portugal

**Keywords:** decision-making, dopamine agonists, risk-taking, rodent models, uncertainty, environmental cues

## Abstract

This paper presents a novel rodent decision-making task that explores uncertainty, independently of expectation and predictability. Using a 5-hole operating box, adult male Wistar rats were given choices between a small certain (safe) food reward and a large uncertain (risk) food reward. We found that animals strongly preferred the safe option when it had a fixed position or was cued with a light in a random placement scheme, but had no preference for safe or risk options when the latter were associated with light. Importantly, when the reward was manipulated animals could perceive alterations in the outcome value and biased their choice pattern to the most profitable option. In addition, we found that the D2/D3 agonist quinpirole biased all decisions toward risk in this paradigm. Finally, a c-fos analysis revealed that several brain areas known to be involved in decision-making mechanisms, including the medial prefrontal cortex, the orbitofrontal cortex, the nucleus accumbens and the striatum, were activated by the task. In summary, this paradigm is a useful and highly reliable tool to explore decision-making processes in contexts of uncertainty.

## Introduction

Making decisions is a common task in our lives that entails evaluation of risks and rewards associated with different options available. When deciding between two goods presented in a different manner, individuals choose based on effort to obtain reward, amount of outcome and chance of win. A growing body of evidence has demonstrated individual differences on choice pattern (Penolazzi et al., 2013) and that proneness to choose high or low risk options are affected by several neuropsychiatric disorders such as schizophrenia (Heerey et al., 2008), obsessive compulsive disorders (Starcke et al., 2010), depression (Smoski et al., 2008), attention deficit and hyperactive disorder (Ernst et al., 2003; Drechsler et al., 2008), addictive disorders (Bechara, 2003) and pathological gambling (Ochoa et al., 2013). Similar observations were obtained using animal paradigms of decision-making that resemble features of those described for humans (for instance, Floresco and Whelan, 2009). However, a strong bias of both animal and human decision-making studies evaluating risk relates to the fact that distinction between high and low uncertainty choices usually also encompasses a decision between advantageous and disadvantageous options, making behavioral analysis more difficult and dubious. Indeed, while any choice possibly, but not certainly, leading to a punishment/loss of reward should be classified as risky, most of these paradigms equate risk with long-term losses (which should not be always the case).

In this regard, one of the most popular paradigms is the rodent equivalent of the Iowa Gambling Task (IGT), developed for humans by Bechara et al. (1994) and adapted for rodents independently by van den Bos et al. (2006b), Pais-Vieira et al. (2007); Rivalan et al. (2009), and Zeeb et al. (2009). In the IGT, the subject has to choose between four options (cards in humans; levers, maze arms or nose poke apertures in rodents), two of which yield higher rewards but also, randomly presented, higher losses than the other two. As a result, choice of the former (disadvantageous options) results in an overall net loss that contrasts with an overall net gain when choosing the latter (advantageous options). Choices in this paradigm depend of the factoring of value, uncertainty and, particularly, time-discount, with near sighted subjects more sensitive to immediate gains than to long-term losses, in what constitutes an interesting model of complex economic decisions. Besides the IGT, other paradigms of risk decision-making for rodents include: (1) risk-discounting tasks (Cardinal and Howes, 2005; Floresco et al., 2008), where subjects have to choose between small certain rewards and large probabilistically delivered rewards presented in a crescent and/or decrescent manner; (2) delay-discounting tasks, characterized by choice between smaller rewards available immediately versus larger rewards available after a varying delay, and frequently used for the study of impulsive choice both in humans (Johnson and Bickel, 2002; Dixon et al., 2003) and in rodents (Ito and Asaki, 1982; Green and Estle, 2003; Kobayashi and Schultz, 2008); (3) risk punishment decision tasks where rats choose between a small safe reward and a large reward associated with punishment (Simon et al., 2007, 2009); (4) effort-discounting tasks (van den Bos et al., 2006a; Floresco et al., 2008; Cocker et al., 2012), evaluating cost/benefit decision-making, where animals choose between a small reward obtainable after a low amount of physical effort and a larger reward after considerably more work.

In fact, available animal models of decision-making, including those specifically designed to assess risk and uncertainty did not isolate uncertainty from value (Jentsch et al., 2010; Winstanley et al., 2011) or do so only in some trials within a single session, in a discounting format (St Onge and Floresco, 2009), precluding a deeper analysis of the neuronal circuits involved and the effects of pharmacological manipulations. As few animal models explore the processing of uncertainty, independently of expectation and predictability, there were three main goals in this study: (i) establishing a new risk-based decision-making paradigm in which rats choose between certain (certain/safe) and uncertain (uncertain/risky options) options, with similar overall expectations and predictability and where animals pattern of choice can be described as neutral in non-manipulated conditions; (ii) mapping the brain regions activated by the task; and (iii) analyze how risk-based decision-making is affected by outcome value manipulations (through increasing or decreasing reward amount) and by a dopaminergic drug that has been previously shown to affect probability based decision-making.

## Materials and methods

### Animals

Sixty adult male Wistar rats (Charles River Laboratories, Barcelona, Spain), aged 2 months and weighting 250–300 g at the start of the experiment, were housed in groups of two under standard laboratory conditions with an artificial light–dark cycle of 12:12 h (lights on from 8:00 A.M. to 8.00 P.M.) in a temperature- and humidity-controlled room. Animals were given 2 weeks to acclimate to the housing conditions with *ad libitum* access to food and water. A food deprivation regimen was initiated 24 h before the initiation of behavioral training and testing to maintain the subjects at approximately 90% of their free-feeding body weight. Rats had free access to water while in the home cage.

All experiments were conducted in accordance with local regulations (European Union Directive 86/609/EEC) and National Institutes of Health guidelines on animal care and experimentation and approved by Direção Geral Veterinária (DGV; the Portuguese National Institute of Veterinary).

### Development of the risk-based decision-making paradigm

Behavioral training and testing took place in square 5-hole operant chambers (OCs, 25 × 25 cm; TSE Systems, Germany). Each chamber has five squared apertures (2.5 cm) mounted into a curved wall and elevated 2 cm from the grid floor, each hole equipped with a light (3 W lamp bulb) and crossed by an infra-red detector that monitored animal nose pokes. In the opposite side, one pellet dispenser is used to deliver rewards into a hole crossed by an infra-red detector to check pellet dispenser entries. Three 5-hole OCs, placed within sound attenuating boxes with individual electrical fans for ventilation and white noise production, were simultaneously used in our studies.

**Figure 1:**
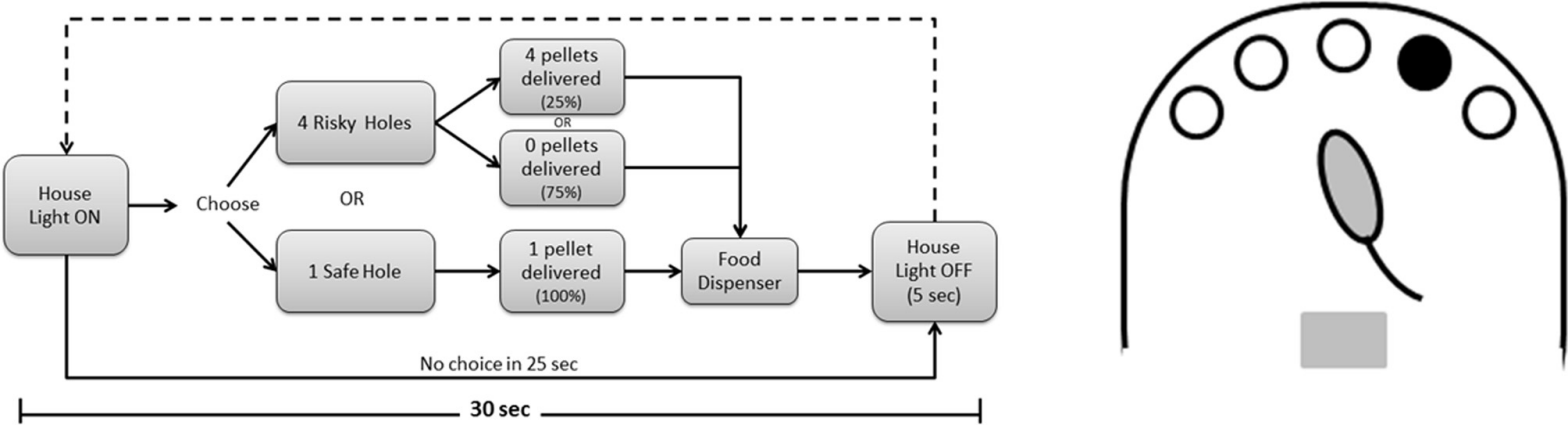
**Risk-taking task.** Flow-chart of one trial in the neutral condition, in which the overall gain is the same for risky or safe choices; each daily session consisted of 100 trials or 30 min of testing.

The decision-making paradigm is presented in Figure 1. Each daily session was initiated by switching the home light on, 5 s after the animal was placed in the chamber, and lasted for 30 min or 100 trials, whichever occurred first. In each trial, rats could choose between a “safe” hole (resulting in the delivery of 1 pellet with 100% probability) and 4 “risk” holes (resulting in the delivery of 4 pellets with 25% probability). In our opinion, this 1 against 4 hole arrangement results in a more naturalistic option that a 1:1 arrangement with the same probabilities. Indeed, in our model the choice is just between playing safe (by nose poking in the non-illuminated hole) or risking (by nose poking in one of the 4 illuminated holes, only one of which will result in reward delivery), the probabilities arising from the number of illuminated holes. Moreover, the use of more holes augments the complexity of the task as well as the possibilities to modulate the gains/losses. Importantly, this design of risky and safe choices evens the overall outcome of either option, allowing an analysis of risk-taking behaviors independently of reward value or delay. After each choice, animals had to check the amount of reward received at the pellet dispenser (they were taught to do it by applying a 10 s “lights off, holes inactive” penalty if they failed to do so), home cage light was switched off and a new trial started 5 s later. Number of trials completed, total time spent, animals' choices and omissions as well as pellets received in each trial were automatically registered by the software and analyzed.

In the process of optimizing the conditions of our risk-based decision-making assessment, we tested three different strategies for cueing risk and safe options using three different sets of animals. Of note, each set of animals was trained for 20 days and choice preferences recorded and analyzed. Our first attempt was to attribute the safe option to one (fixed) hole, with the five different positions being evenly distributed among different animals to even out any placement bias (fixed placement condition.). As this resulted in a strong bias toward the safe option (see the results section), that hampered the observation of minor shifts in behavior, we used a different group of animals to test a second condition in which the safe option was signalized with a light and randomly attributed, in each trial, to one of the 5 holes (random placement—light safe condition). Curiously, this also resulted in a strong preference for the safe choices (see the results section), which made us test a third, and final condition, in which the safe option was signalized with the absence of light (the risk option all had a light on) and randomly attributed, in each trial, to one of the 5 holes (random placement—light risk condition). Importantly, this was the condition used in all our experiments thereon, namely those described in the remainder of the present paper.

In all subsequent experiments, animals were trained in this final protocol (“random placement—light risk”) and tested in 3 consecutive 8-day decision-making paradigms: in the first, safe and risk choices were rewarded with 1 and 4 pellets, respectively, as described above (Figure 1), resulting in no net gain (neutral condition); in the second, only the risk choice reward was doubled (8 instead of 4), resulting in an average long-term profit for those who risk (risk favorable condition); in the third, only the reward in safe choices was doubled (2 instead of 1), resulting in a long-term profit for those who tend to choose safe (safe favorable condition).

### C-fos immunohistochemistry

A separate set of 10 animals were trained in the “random placement—light risk” paradigm until acquisition of the task (10 days) and then tested in the neutral condition for an additional 10 days. In the last day of testing, animals were sacrificed 90 min after the end of the behavioral task with a lethal injection with pentobarbital and then transcardially perfused with phosphate buffered saline (PBS) followed by 4% paraformaldehyde (PFA). Control animals were exposed to the same conditions, but in the last day of testing were rewarded in the OC independently of nose poking, in an overall amount similar to that of the tested animals. Brains were removed and post-fixed in PFA for 4 h and then transferred to an 8% sucrose solution and kept at 4°C. 50 μm coronal sections of the forebrain were serially cut on a vibratome at 50 μm and collected in PBS (0.1 M; pH7.2). For c-fos immunohistochemistry, sections were firstly incubated in H2O2 (3.3% in PBS) solution for 30 min and then sequentially washed in PBS and PBS-T (0.3% triton X-100; Sigma-Aldrich). Sections were then incubated in 2.5% (in PBS-T) fetal bovine serum for 2 h followed by anti-fos primary antibody [1:2000 in the same solution; PC38 Anti-c-Fos (Ab-5), Calbiochem] overnight. After several washes in PBS-T, sections were incubated with secondary antibody (1:200 in PBS-T; polyclonal swine anti-rabbit E0353, DAKO) for 1 h, again washed in PBS-T and incubated in avidin-biotin complex (ABC, 1:200, Vector Laboratories) for 1 h. Sections were then sequentially washed with PBS-T, PBS and Tris-HCl (0.05 M, pH 7.6) and incubated in 0.0125% diaminobezidine tetrahydrochloride (DAB; Sigma Immunochemicals, St. Louis, USA) and 0.02% H2O2 in Tris-HCl for 3–5 min to reveal the labeling. Finally, sections were placed on SuperFrost Plus slides (Braunschweig, Germany), dehydrated and counterstained with hematoxylin. All procedures were performed at room temperature.

The number of c-fos positive cells was counted within the boundaries of the medial prefrontal cortex [prelimbic cortex (PrL), infralimbic cortex (IL) and cingulate cortex (Cg1)], orbitofrontal cortex [medial (MO), ventral (VO) and lateral (LO) parts], somatosensory cortex (SSC), motor cortex (MC), insula, dorsal striatum [dorsolateral striatum (DLS) and dorsomedial striatum (DMS)], and nucleus accumbens [shell (NAcS) and core (NAcC)] as defined by the Paxinos and Watson (1998). c-fos positive cells densities (number of positive cells/cross sectional area of the region of interest) were calculated for comparisons between groups. Cross sectional area of each region was calculated according to the Cavalieri principle (Gundersen et al., 1988). For this, we randomly superimposed onto each area a test point grid in which the interpoint distance, at tissue level, was: 100 μm for IL and MO; 150 μm for PL, VO and LO; 350 μm for MC, SSC, NAcS and NAcC; and 500 μm for DLS and DMS, and counted the points that fell into the boundaries of the region of interest. These procedures were done using using StereoInvestigator software (MBL Neuroscience, VT) and a camera attached to a motorized microscope.

### Treatment with the D2/D3 agonist

A separate set of 20 animals was trained in the “random placement—light risk” paradigm for 10 days and then tested in the neutral, risk favorable and safe favorable conditions (8 days in each). In the last 3 days of each test, half of the animals received injections of the dopamine D2/D3 agonist quinpirole while others received vehicle. Quinpirole hydrochloride (0.15 mg/kg; Sigma-Aldrich), dissolved in 0.9% sterile saline to a volume of 1 ml/Kg, was administered intraperitoneally. Injections were given 15 min before behavioral testing and dose was selected in accordance with previous reports showing behavioral effects of the drug (Kurylo and Tanguay, 2004; Boulougouris et al., 2009).

### Statistical analysis

Data was analyzed using SPSS (version 19.0; IBM). Results are expressed as group means ± SE. Differences between groups were analyzed using independent-samples Student's *t*-test (for c-fos activation) and repeated measures ANOVA (for behavioral data). Differences were considered to be significant if *p* < 0.05.

## Results

**Figure 2:**
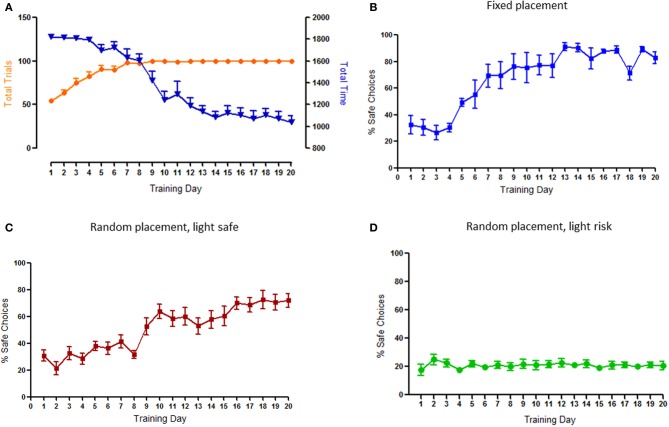
**Behavioral characterization of the task and effects of environmental cue modeling on risk-based decision. (A)** Total time and number of trials by session. Animals significantly decreased the total session time and increased the number of trials per session until the maximum of 100 in the first 2 weeks. **(B)** Pattern of choices using a fixed placement of the safe nose-poke hole. Animals increase their preference for safe choices to more than 80%. **(C)** Pattern of choices when the only illuminated nose-poke hole was the safe/certain option—light signaled safe. Animals consistently increased their preference for this option to more than 60%. **(D)** Pattern of choices when the nose-poke holes corresponding to risk/uncertain options were illuminated. Animals stabilize their performance at around 20% of safe choices (choice levels), without a net preference for risk or safe. This was the design adopted in the final version of the task.

As expected, during training animals increased the number of completed trials in each session, inversely decreasing total time spent to do so; by the 8th day of training all animals were able to complete the maximum number of trials (100) (Figure 2A).

As already mentioned, the first set of experiments was devoted to searching the appropriate cueing for this risk-based decision-making task. When the safe option was fixed in the same hole during the entire protocol, animals rapidly acquired (from the 5th day) and maintained a strong (>80%) preference for this option (Figure 2B). Similarly, when the safe option was randomly placed but associated with a light, animals had a clear (>60%) preference for safe choices, that was evident from the 9th day of training (Figure 2C). On the contrary, when the safe option was randomly placed but associated with absence of light, animals did not display any preference between safe and risk options, a pattern of choices that was established relatively early and maintained during the entire protocol (Figure 2D). The latter design was selected for the final version of the task and used in the subsequent analysis.

Having set the task design, we explored the brain areas activated by its performance by analyzing c-fos expression, an immediate early gene whose expression is triggered by neuronal activation. In comparison with animals rewarded independently of nose poking (controls), in an overall amount similar to that of the tested animals, performance of the task in the neutral condition induced the activation of several brain areas, including the medial prefrontal cortex subregions (PrL: *t* = −3.61, *P* < 0.05; IL: *t* = −1.58, *P* < 0.05; Cg1: *t* = −2.22, *P* < 0.05), orbitofrontal cortex subregions (Medial OFC: *t* = −3.14, *P* < 0.05; Ventral OFC: *t* = −3.97, *P* < 0.05; Lateral OFC: *t* = −7.28, *P* < 0.05), insular cortex (*t* = −4.45, *P* < 0.05), dorsal striatum (DLS: *t* = −5.45, *P* < 0.05; DMS: *t* = −2.86, *P* < 0.05), and nucleus accumben*s* (NAcc Shell: *t* = −2.41, *P* < 0.05; NAcc Core: *t* = −3.76, *P* < 0.05). No differences were found in the activation of the principal somatosensory (*t* = −0.88, *P* = 0.40) and motor cortices (*t* = −1.78, *P* = 0.09) (Table 1).

**Table 1:** **c-fos positive cells by region**.

**Brain region**	**Operant chamber controls (*n* = 10)**	**Risk-based decision-making task (*n* = 10)**	**Statistics**
LOFC	2.27 ± 0.39	6.23 ± 0.38	*t* = −7.28^*^
VOFC	3.66 ± 0.60	8.60 ± 1.09	*t* = −3.97^*^
MOFC	2.81 ± 0.59	6.00 ± 0.82	*t* = −3.14^*^
Ins	0.79 ± 0.21	2.02 ± 0.18	*t* = −4.45^*^
PrL	2.88 ± 0.54	5.29 ± 0.40	*t* = −3.61^*^
IL	1.53 ± 0.25	5.39 ± 0.20	*t* = −1.58^*^
Cg1	2.63 ± 0.60	5.80 ± 0.96	*t* = −2.22^*^
NaccC	2.04 ± 0.52	4.90 ± 0.56	*t* = −3.76^*^
NaccS	2.69 ± 0.65	4.64 ± 0.47	*t* = −2.41^*^
DLS	1.02 ± 0.23	4.08 ± 0.51	*t* = −5.45^*^
DMS	1.76 ± 0.36	4.25 ± 0.80	*t* = −2.86^*^
SSC	2.09 ± 0.28	2.54 ± 0.42	*t* = −0.88
MC	2.07 ± 0.08	2.71 ± 0.34	*t* = −1.78

Values in cells/μm^2^ (x1000) ± s.e.m.; L/V/MOFC, lateral/ventral/medial OFC; PrL, Prelimbic cortex; IL, infralimbic cortex; Cg1, cingulate cortex; NaccC/S, Nacc Core/Shell; DLS/DMS, Dorsomedial/lateral striatum; SSC, somatosensorial cortex; MC, motor cortex. Controls were placed in the operant chamber and rewarded the same amount of sucrose pellets, but independently of nose-poking.

* p < 0.05 vs. similar reward no-contingency controls.

**Figure 3:**
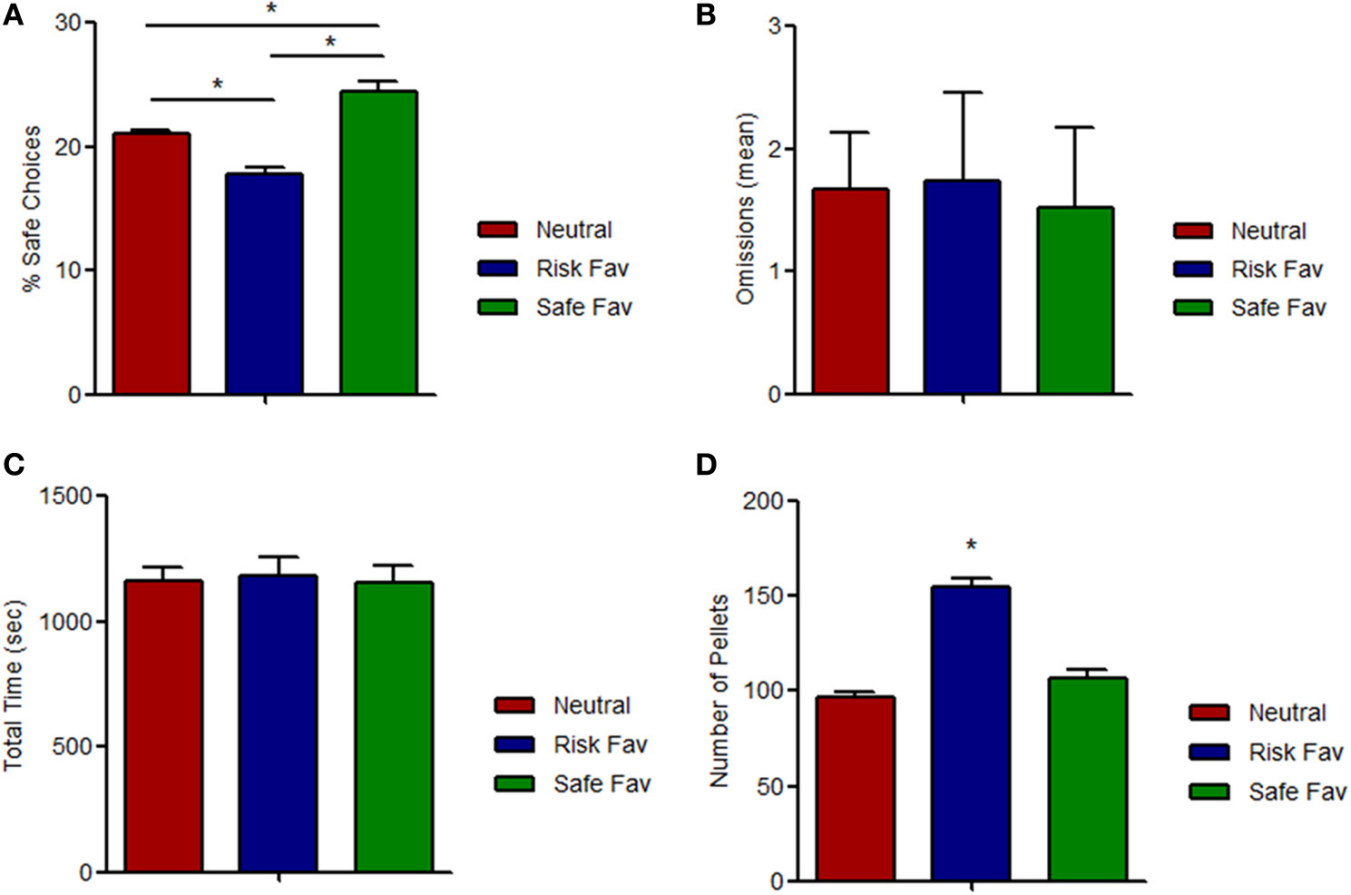
**Effects of outcome value manipulation on risk-based decision. (A)** Behavioral responses to outcome value manipulations. Increases (doubling) in the amount of reward of the risky choices (risk favorable condition) or the safe choices (safe favorable condition) lead to a reduction (risk favorable) or increase (safe favorable) in the % of safe choices, compared with the neutral condition, which is of similar magnitude. **(B)** Average number of omissions per each daily session. No differences were found between conditions. **(C)** Total time spent by each daily session did not change among different paradigms. **(D)** Total pellets received by daily session. Significant differences were found on risk favorable paradigm. ^*^p < 0.05.

We then set to assess whether preference for safe choices could be manipulated, and first tested the impact of changes in reward magnitude. When rewards for either the risk or the safe options were increased (risk favorable or safe favorable conditions, respectively), animals switched their pattern of choices accordingly, decreasing (−15.3% ± 6.68), or increasing (+16.0% ± 8.70) the percentage of safe choices relative to the baseline (condition: *F* = 73.928, *p* < 0.001) (Figure 3A). No differences were found among omissions (condition: *F* = 0.055, *p* = 0.947) (Figure 3B) and total time spent (condition: *F* = 0.069, *p* = 0.933) (Figure 3C) in the three different paradigms. As expected, the number of total pellets received while in the risk favorable condition was higher than in the other two conditions (condition: *F* = 66.867, *p* < 0.001) (Figure 3D).

**Figure 4:**
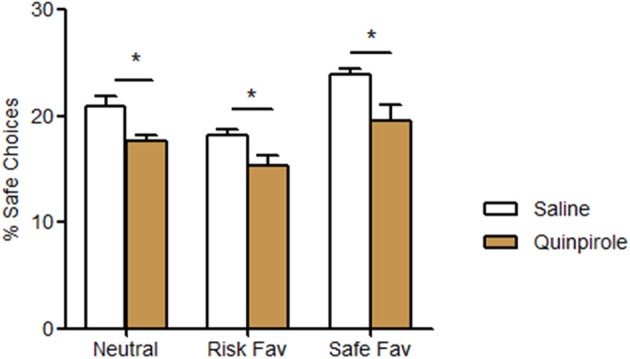
**Acute behavioral effects of D2/D3 agonist Quinpirole.** Quinpirole treated animals increased the percentage of risk choices in all different probabilities protocols. ^*^p < 0.05.

In our last experiment, we assessed the impact of quinpirole, a D2/D3 agonist known to influence decision-making strategies, in the performance of our task. Quinpirole-treated animals displayed lower rates of safe choices (neutral −17.7%, risk favorable −18.4%, safe favorable −22.3%) in all testing conditions when contrasted to controls (Figure 4; treatment *F* = 23.101, *p* < 0.001; condition *F* = 22.765, *p* < 0.001; interaction *F* = 1.217, *p* = 0.308). Despite these diminished rate of safe choices, they keep changing their pattern of choices among the three different conditions (neutral, risk favorable and safe favorable). No differences were found among the number of total pellets received, omissions and total time spent between treated and non-treated animals (data not shown).

## Discussion

Given the growing interest in neuroeconomics, several experimental paradigms of gambling and/or risky decision-making in rats have been put forward in the past years, with the aim of studying such behaviors in animal model and facilitating the dissection of the neural substrate of economic decisions and its modulators.

Through recent years, several animal paradigms were developed to measuring decision-making when the choice is based on a context of conflict related to one or more of the following components: the probability, the effort, the delay and the risk of punishment. Importantly, none was designed to isolate uncertainty from value, effort or time-discounting. Even the paradigms specifically developed to the assessment of risky decision-making such as the Rodent Version of the balloon Analog Risk Task (Jentsch et al., 2010) were not suited to evaluate uncertainty, emphasizing on the amount of risk that subjects are willing to accept to obtain a reward. In order to specifically address this determinant of decision-making we developed a novel risk-based task. In it, animals have to choose, by making a nose poke, between a non-illuminated hole that always triggers the delivery of a reward (certain/safe option) and four illuminated holes, only one of which will trigger the delivery of a 4 times bigger reward (uncertain/risky options), in what amounts to a 25% probability. Importantly, due to this design, both choices yield, on the long run, the same amount of reward, thus isolating uncertainty from both value and time-discounting. Additionally, as probability of win in the uncertain/risk option is kept constant, uncertainty is isolated from amount of risk. This, in our opinion, represents a major advantage of our task.

Another modulator of animals' behavior addressed in the preliminary tests leading to the final design was the presence of light. In this regard, we found that, associating a light with the hole corresponding with the safe choice resulted in a clear preference for safe options, whereas signaling the risky options with light (and the safe hole with the absence of light) resulted in a balanced behavior in which animals chose each option approximately at chance levels (20%). Since they choose each option at chance levels, it could be argued that animal choices were random. However, this is not likely to happen since animals changed their behavior to the most profitable option when paradigm was adapted to favor risk or safe. Interestingly, the fact that, in basal conditions, animals have a similar preference for the safe and each of the risk options is also a distinctive aspect of our task in respect to previous ones in as much as it facilitates the study of risky-behavior modulators, including manipulations of reward or timing, drug treatments or environmental factors.

In order to test this possibility in of our paradigm, we decided to manipulate the value of each option, and found that animals were able to recognize such changes and shift their preference accordingly, as revealed by an increased preference to risky options when risk profit was doubled and to safe options when amount of reward was increased. Importantly, we also found that acute administration of D2/D3 agonist quinpirole biases behavior to risk, which was in accordance with previously reported effects of dopaminergic agents on decision-making behaviors, associating dopaminergic agonists with increased rates of risk choices (Riba et al., 2008; St Onge and Floresco, 2009; St Onge et al., 2010). These observation could raise three different explanations, all possibly triggered by an augmented dopaminergic tone: first, this bias can be related with an overestimation of probabilities associated with risk options; second, it might involves an increase in random choices or the establishment of habitual perseverative behaviors; third, it can be related with an increased preference for light-associated choices. Interestingly, these animals were still able to adapt their choices upon changes in outcome value. Indeed, despite of displaying higher rates of safe choices than controls even when it was less profitable, quinpirole treated animals were able to update their representation of the relative value of each option. These mechanisms seem to be mediated by a fronto-striato-thalamic-frontal circuit that involves mPFC, OFC and dorsal striatum, areas found to be activated by the task. Interestingly, contradictory data emerged from previous reports on the mPFC contribution to risk-based decision-making: while St Onge et al. (2011) described a disruption of risk-based decisions induced by quinpirole and an increasing of risky behavior induced by D2 specific antagonist eticlopride, both specifically injected on the mPFC, other studies report that mPFC inactivation (D2 receptors are inhibitory) as well as disruption of communication with basolateral amygdala is associated with decreased risk aversion (St Onge and Floresco, 2009; St Onge et al., 2012). Additionally, the OFC was found necessary to the increased expression of incentive motivation to obtain larger rewards (Jentsch et al., 2010), which allows us to speculate that this brain region was necessary to the expression of quinpirole-induced risk-prone behaviors. In line with our results, striatal D2/D3 receptors were also found to mediate risk-based decisions with D2/D3 antagonists promoting a decreased rate of uncertain choices in rats with lower striatal levels of these receptors (Cocker et al., 2012).

Finally, we characterized the brain activation patterns recruited by our task and found increased activity (as assessed by increased c-fos expression) in almost all key areas known to be involved in decision-making processes, including the OFC and mPFC, the insular cortex, the dorsal striatum and the nucleus accumbens. Interestingly, these areas were also shown to be engaged in performance of the IGT including the ventro-medial prefrontal cortex (Bechara et al., 1999; Fellows and Farah, 2005), the dorsolateral prefrontal cortex (Manes et al., 2002; Bolla et al., 2004; Fellows and Farah, 2005), the orbitofrontal cortex (Manes et al., 2002; Bolla et al., 2004; Hsu et al., 2005), the anterior cingulate cortex (ACC) (Tucker et al., 2004) and the striatum (Hsu et al., 2005). Such similarities are not surprising since, despite differences in task design, both require the processing of uncertainty, the representation of value and the prediction of reward, three of the main components of decision-making which have been mapped, respectively, to the loop between the NAcc and the OFC (Doya, 2008), the OFC and ACC (Tremblay and Schultz, 1999; Gehring and Willoughby, 2002; Padoa-Schioppa and Assad, 2006) and the dorsal striatum (Schultz, 2002). However, these results should be carefully analyzed since we used as controls animals passively receiving food on the operate behavior chamber. In this regard, use of a better control, such as animals on a task with 5 “safe” holes, could improve the robustness of our findings.

Altogether, these features suggest that this novel behavioral paradigm is valuable in exploring animal preferences in a context of uncertainty/risk, independently of value, effort, amount of risk, expectation and predictability. The ability to isolate different components of the decision-making process is of relevance to better understand the conditions in which these processes are impaired and, eventually, to better define intervention strategies that might remediate impairments on decisions.

### Conflict of interest statement

The authors declare that the research was conducted in the absence of any commercial or financial relationships that could be construed as a potential conflict of interest.

## References

[B1] Bechara A. (2003). Risky business: emotion, decision-making, and addiction. J. Gambl. Stud. 19, 23–51 10.1023/A:1021223113233 12635539

[B2] Bechara A. Damasio A. R. Damasio H. Anderson S. W. (1994). Insensitivity to future consequences following damage to human prefrontal cortex. Cognition 50, 7–15 10.1016/0010-0277(94)90018-3 8039375

[B3] Bechara A. Damasio H. Damasio A. R. Lee G. P. (1999). Different contributions of the human amygdala and ventromedial prefrontal cortex to decision-making. J. Neurosci. 19, 5473–5481 10377356 10.1523/JNEUROSCI.19-13-05473.1999 PMC6782338

[B4] Bolla K. I. Eldreth D. A. Matochik J. A. Cadet J. L. (2004). Sex- related differences in a gambling task and its neurological correlates. Cereb. Cortex 14, 1226–1232 10.1093/cercor/bhh083 15142963

[B5] Boulougouris V. Castañé A. Robbins T. W. (2009). Dopamine D2/D3 receptor agonist quinpirole impairs spatial reversal learning in rats: investigation of D3 receptor involvement in persistent behavior. Psychopharmacology (Berl.) 202, 611–620 10.1007/s00213-008-1341-2 18836703

[B6] Cardinal R. N. Howes N. J. (2005). Effects of lesions of the nucleus accumbens core on choice between small certain rewards and large uncertain rewards in rats. BMC Neurosci. 6:37 10.1186/1471-2202-6-37 15921529 PMC1177958

[B7] Cocker P. J. Dinelle K. Kornelson R. Sossi V. Winstanley C. A. (2012). Irrational choice under uncertainty correlates with lower striatal D(2/3) receptor binding in rats. J. Neurosci. 32, 15450–15457 10.1523/JNEUROSCI.0626-12.2012 23115182 PMC6621583

[B8] Dixon M. R. Marley J. Jacobs E. A. (2003). Delay discounting by pathological gamblers. J. Appl. Behav. Anal. 36, 449–458 10.1901/jaba.2003.36-449 14768665 PMC1284461

[B9] Doya K. (2008). Modulators of decision making. Nat. Neurosci. 11, 410–416 10.1038/nn2077 18368048

[B10] Drechsler R. Rizzo P. Steinhausen H. C. (2008). Decision-making on an explicit risk-taking task in preadolescents with attention-deficit/hyperactivity disorder. J. Neural Transm. 115, 201–209 10.1007/s00702-007-0814-5 17885724

[B11] Ernst M. Kimes A. S. London E. D. Matochik J. A. Eldreth D. Tata S. (2003). Neural substrates of decision making in adults with attention deficit hyperactivity disorder. Am. J. Psychiatry 160, 1061–1070 10.1176/appi.ajp.160.6.1061 12777263

[B12] Fellows L. K. Farah M. J. (2005). Different underlying impairmentsin decision-making following ventro- medial and dorsolateral frontal lobe damageinhumans. Cereb.Cortex 15, 58–63 10.1093/cercor/bhh108 15217900

[B13] Floresco S. B. Tse M. T. Ghods-Sharifi S. (2008). Dopaminergic and glutamatergic regulation of effort- and delay-based decision making. Neuropsychopharmacology 33, 1966–1979 10.1038/sj.npp.1301565 17805307

[B14] Floresco S. B. Whelan J. M. (2009). Perturbations in different forms of cost/ benefit decision making induced by repeated amphetamine exposure. Psychopharmacology 205, 189–201 10.1007/s00213-009-1529-0 19365622

[B15] Gehring W. J. Willoughby A. R. (2002). The medial frontal cortex and the rapid processing of monetary gains and losses. Science 295, 2279–2282 10.1126/science.1066893 11910116

[B16] Green L. Estle S. J. (2003). Preference reversals with food and water reinforcers in rats. J. Exp. Anal. Behav. 79, 233–242 10.1901/jeab.2003.79-233 12822689 PMC1284932

[B17] Gundersen H. J. Bendtsen T. F. Korbo L. Marcussen N. Moller A. Nielsen K. (1988) Some new, simple and efficient stereological methods and their use in pathological research and diagnosis. APMIS 96, 379–394 10.1111/j.1699-0463.1988.tb05320.x 3288247

[B18] Heerey E. A. Bell-Warren K. R. Gold J. M. (2008). Decision-making impairments in the context of intact reward sensitivity in schizophrenia. Biol. Psychiatry 64, 62–69 10.1016/j.biopsych.2008.02.015 18377874 PMC2613513

[B19] Hsu M. Bhatt M. Adolphs R. Tranel D. Camerer C. F. (2005).Neural systems responding to degrees of uncertainty in human decision- making. Science 310, 1680–1683 10.1126/science.1115327 16339445

[B20] Ito M. Asaki K. (1982). Choice behavior of rats in a concurrent-chains schedule: amount and delay of reinforcement. J. Exp. Anal. Behav. 37, 383–392 10.1901/jeab.1982.37-383 16812274 PMC1333154

[B21] Jentsch J. D. Woods J. A. Groman S. M. Seu E. (2010). Behavioral characteristics and neural mechanisms mediating performance in a rodent version of the Balloon Analog Risk Task. Neuropsychopharmacology 35, 1797–1806 20375994 10.1038/npp.2010.47 PMC3055471

[B22] Johnson M. W. Bickel W. K. (2002). Within-subject comparison of real and hypothetical money rewards in delay discounting. J. Exp. Anal. Behav. 77, 129–146 10.1901/jeab.2002.77-129 11936247 PMC1284852

[B23] Kobayashi S. Schultz W. (2008). Influence of reward delays on responses of dopamine neurons. J. Neurosci. 28, 7837–7846 10.1523/JNEUROSCI.1600-08.2008 18667616 PMC3844811

[B24] Kurylo D. D. Tanguay S. (2004). Effects of quinpirole on behavioral extinction. Physiol. Behav. 80, 1–7 10.1016/S0031-9384(03)00218-X 14568302

[B25] Manes F. Sahakian B. Clark L. Rogers R. Antoun N. Aitken M. (2002). Decision- making processes following damage to the prefrontal cortex. Brain 125, 624–639 10.1093/brain/awf049 11872618

[B26] Ochoa C. Alvarez-Moya E. M. Penelo E. Aymami M. N. Gómez-Peña M. Fernández-Aranda F. (2013). Decision-making deficits in pathological gambling: the role of executive functions, explicit knowledge and impulsivity in relation to decisions made under ambiguity and risk. Am. J. Addict. 22, 492–499 10.1111/j.1521-0391.2013.12061.x 23952896

[B27] Padoa-Schioppa C. Assad J. A. (2006). Neurons in the orbitofrontal cortex encode economic value. Nature 441, 223–226 10.1038/nature04676 16633341 PMC2630027

[B28] Pais-Vieira M. Lima D. Galhardo V. (2007). Orbitofrontal cortex lesions disrupt risk assessment in a novel serial decision- making task for rats. Neuroscience 145, 225–231 10.1016/j.neuroscience.2006.11.058 17204373

[B28a] Paxinos G. Watson C. (1998). The Rat Brain in Stereotaxic Coordinates. San Diego, CA: Academic Press

[B29] Penolazzi B. Leone L. Russo P. M. (2013). Individual differences and decision making: when the lure effect of gain is a matter of size. ONE 8:e58946 10.1371/journal.pone.0058946 23484058 PMC3590131

[B30] Riba J. Kramer U. M. Heldmann M. Richter S. Munte T. F. (2008). Dopamine agonist increases risk taking but blunts reward-related brain activity. PLoS ONE 3:e2479 10.1371/journal.pone.0002479 18575579 PMC2423613

[B31] Rivalan M. Ahmed S. H. Dellu-Hagedorn F. (2009). Risk-prone individuals prefer the wrong options ona rat version of the Iowa gambling task. Biol. Psychiatry 66, 743–749 10.1016/j.biopsych.2009.04.008 19482266

[B32] Schultz W. (2002). Getting formal with dopamine and reward. Neuron 36, 241–263 10.1016/S0896-6273(02)00967-4 12383780

[B33] Simon N. W. Gilbert R. J. Mayse J. D. Bizon J. L. Setlow B. (2009). Balancing risk and reward: a rat model of risky decision making. Neuropsychopharmacology 34, 2208–2217 10.1038/npp.2009.48 19440192 PMC2726909

[B34] Simon N. W. Mendez I. A. Setlow B. (2007). Cocaine exposure causes long term increases in impulsive choice. Behav. Neurosci. 121, 543–549 10.1037/0735-7044.121.3.543 17592945 PMC2581406

[B35] Smoski M. J. Lynch T. R. Rosenthal M. Z. Cheavens J. S. Chapman A. L. Krishnan R. R. (2008). Decision-making and risk aversion among depressive adults. J. Behav. Ther. Exp. Psychiatry 39, 567–576 10.1016/j.jbtep.2008.01.004 18342834 PMC2590786

[B36] Starcke K. Tuschen-Caffier B. Markowitsch H. J. Brand M. (2010). Dissociation of decisions in ambiguous and risky situations in obsessive-compulsive disorder. Psychiatry Res. 175, 114–120 10.1016/j.psychres.2008.10.022 20004479

[B37] St Onge J. R. Abhari H. Floresco S. B. (2011). Dissociable contributions by prefrontal D1 and D2 receptors to risk-based decision making. J. Neurosci. 31, 8625–8633 10.1523/JNEUROSCI.1020-11.2011 21653866 PMC6623317

[B38] St Onge J. R. Chiu Y. C. Floresco S. B. (2010). Differential effects of dopaminergic manipulations on risky choice. Psychopharmacology 211, 209–221 10.1007/s00213-010-1883-y 20495787

[B39] St Onge J. R. Floresco S. B. (2009). Dopaminergic modulation of risk- based decision making. Neuropsychopharmacology 34, 681–697 10.1038/npp.2008.121 18668030

[B40] St Onge J. R. Stopper C. M. Zahm D. S. Floresco S. B. (2012). Separate prefrontal-subcortical circuits mediate different components of risk-based decision making. J. Neurosci. 32, 2886–2899 10.1523/JNEUROSCI.5625-11.2012 22357871 PMC3609657

[B41] Tremblay L. Schultz W. (1999). Relative reward preference in primate orbitofrontal cortex. Nature 398, 704–708 10.1038/19525 10227292

[B42] Tucker K. A. Potenza M. N. Beauvais J. E. Browndyke J. N. Gottschalk P. C. Kosten T. R. (2004). Perfusion abnormalities and decision making in cocaine dependence. Biol. Psychiatry 56, 527–530 10.1016/j.biopsych.2004.06.031 15450790

[B44] van den Bos R. Lasthuis W. Den Heijer E. Van Der Harst J. Spruijt B. (2006b). Toward a rodentmodel of the Iowa gambling task. Behav. Res. Methods 38, 470–478 10.3758/BF03192801 17186757

[B43] van den Bos R. van der Harst J. Jonkman S. Schilders M. Spruijt B. (2006a). Rats assess costs and benefits according to an internal standard. Behav. Brain Res. 171, 350–354 10.1016/j.bbr.2006.03.035 16697474

[B45] Winstanley C. A. Cocker P. J. Rogers R. D. (2011). Dopamine modulates reward expectancy during performance of a slot machine task in rats: evidence for a “near-miss” effect. Neuropsychopharmacology 36, 913–925 10.1038/npp.2010.230 21209612 PMC3077261

[B46] Zeeb F. D. Robbins T. W. Winstanley C. A. (2009) Serotonergic and dopaminergic modulation of gambling behavior as assessed using a novel rat gambling task. Neuropsychopharmacology 34, 2329–2343 10.1038/npp.2009.62 19536111

